# Understanding adolescent girls’ thoughts and opinions on having social media influencers deliver body image and mental health support: A mixed-methods study

**DOI:** 10.1177/20552076251361340

**Published:** 2025-08-03

**Authors:** Sharon Haywood, Phillippa C Diedrichs, Nicole Paraskeva

**Affiliations:** 1 University of the West of England, Centre for Appearance Research, Bristol, UK

**Keywords:** Adolescence, influencers, body image, mental health, interventions, adolescent girls, mixed methods, UK

## Abstract

**Objective:**

Influencers have been used to deliver messaging in public health campaigns and have begun collaborating with researchers to deliver evidence-based content. Building on this approach, this study explored adolescent girls’ perspectives on influencers and utilising influencers to deliver body image and mental health interventions.

**Methods:**

A mixed-methods study was conducted with 375 UK-based girls (*M_age_*  =  15.39, *SD *= 1.63) to understand their experiences with influencers and their opinions about influencer-delivered body image and mental health interventions. Data were analysed quantitatively via descriptive statistics and qualitatively using inductive content analysis.

**Results:**

Most participants expressed likeability, credibility, authenticity, and relatability were key influencer qualities when choosing to trust an influencer, whilst their ethnicity, gender, and body size were not important. In considering intervention development, the most salient factor in trusting an influencer about body image was that the influencer shared their own experiences. Participants preferred offline body image support (47%) versus online (38%); however, just over 60% agreed that influencer-delivered body image interventions were a good idea. The content analysis generated three main categories: ‘Influencer-delivered interventions would be helpful’ (53.5%), encompassing the offer of support and online environment as ideal; ‘influencer-delivered interventions would be unhelpful’ (28.1%), including influencers are not qualified and the toxicity of the internet; and ‘influencer qualities’ (18.3%), such as relatability.

**Conclusion:**

Overall, adolescent girls thought an influencer-driven approach could be helpful in improving their body image and mental health. Caveats included that the influencer must be trustworthy, relatable, and authentic, which could encompass self-disclosure related to their mental health.

## Introduction

Adolescence is a developmental period of great consequence, particularly pertaining to mental health, in that global evidence indicates more than half of all mental disorders that persist throughout the lifespan commence by mid-adolescence,^1,2^ such as depression, anxiety, eating disorders, and substance use disorders.^3^ The World Health Organization reports 14% of young people between the ages of 10 and 19 are afflicted,^4^ compounded by a lack of treatment,^4,5^ which has amounted to a global mental health crisis exacerbated by the COVID-19 pandemic.^6^ Notably, a higher incidence of adolescent girls as compared to boys are impacted by certain disorders, particularly depression and anxiety.^6,7^ Investigations by the National Health Service (NHS) in England found that over 20% of girls aged 11 to 16 are likely to struggle with mental health issues,^8^ growing to almost 32% for 17- to 19-year-old adolescent girls,^8^ which is over double what boys in the same age range experience.^8^ Critically, a mental health concern that disproportionately impacts adolescent girls and has far-reaching health and wellbeing consequences is negative body image.^9,10^

Negative body image or body dissatisfaction – experiencing negative feelings, thoughts, and/or behaviours about one's appearance^11^ – is far from benign. Prospective research shows it is a predictor for an array of negative psychological outcomes, such as depression,^12^ low self-esteem,^13^ self-harm,^12^ and eating disorders,^14^ in addition to high-risk health behaviours, such as unsafe sex practices,^12^ smoking,^12^ and increased alcohol consumption.^12^ Additionally, body dissatisfaction has implications for physical health in that it predicts exercise avoidance^15^ and lower consumption of fruits and vegetables.^16^ Given the critical role this linchpin risk factor plays in the mental and physical health of adolescents, particularly girls,^9,10^ there is an urgent need for an evidence-based interventional approach to mitigate body dissatisfaction at scale.^17^ To date, body image interventions for this target group are predominantly school based,^18,19^ in addition to some community and clinical environments.^20^ These interventions have generally shown to improve body image with small effect sizes in the short term,^19,20^ but wide-scale implementation is difficult.^21^ Barriers include the limited availability of professionals to deliver the programmes, high running costs, and geographical obstacles, such as with rural dwellers.^22,23^ To add to these barriers, unfortunately adolescents in need of mental health support often do not seek help, especially those who require it the most.^24^ The most salient obstacle to help-seeking by young people is the stigma attached to mental health issues,^25^ which relates closely to adolescents’ desire for privacy and anonymity.^23^ A potential solution to addressing these barriers and delivering body image and other mental health interventions to adolescents at scale is to adopt an online format.^21^ A systematic review exploring the use of online mental health services among young people identified that the internet reduces key barriers as there are no geographical restrictions to access it; costs are low to non-existent; and its anonymous nature minimises stigma and embarrassment.^26^ Specifically, meeting young people where they’re at – social media – is a promising avenue to explore.^2,23^

Social media use among young people is ubiquitous. For instance, in the United Kingdom, 93% of 12- to 15-year-olds and 97% of 16- and 17-year-olds use social media,^27^ and in the United States, adolescents spend an average of almost 5 hours per day on social media.^28^ Specifically, YouTube is the most popular social media platform among young people, with 88% in the United Kingdom^29^ and 90% in the United States,^30^ followed by TikTok (53% in the United Kingdom^29^ and 63% in the United States^30^). They commonly turn to social media for information about mental health,^31^ but they also question how reliable such information is.^32^ Indeed, evidence confirms that health misinformation on social media is commonplace.^33^ Additionally, as social media use is associated with negative body image and mental health concerns,^34,35^ infiltrating this space with scientifically informed tools could potentially work to counteract this negative impact.^34,36^ Taken together, there is a clear need for evidence-based resources adolescents can trust.^37^ Although utilising social media as an intervention-delivery mechanism in adolescent mental health is in its infancy, a recent scoping review of social media-based mental health interventions for adolescents and young adults found evidence to support this innovative approach in improving mental health outcomes.^38^ As well, a systematic review examining the use of social media in mental health interventions showed that young people find this approach to be highly acceptable due to ease of use and being housed in a format they find engaging and supportive.^39^ Regarding body image interventions embedded in social media aimed at adolescents, to our knowledge, there are only two – a video-based series and a chatbot – both of which demonstrated improvements in state- and trait-based body image.^40,41^ Given the positive preliminary evidence of acceptability and effectiveness of social media-based interventions to improve body image and other mental health concerns among adolescents, a relatively untapped area within the context of social media deserving of exploration is the use of influencers.^42^

Influencers create engaging content on social media platforms such as YouTube, TikTok, and Instagram for their loyal followers, who range in the thousands to millions.^43^ Their content, which may or may not be company sponsored or paid, can persuade and impact the attitudes and behaviours of their regular viewers, many of whom are adolescents.^44–46^ The power of influencers can be interpreted through the theoretical framework of the two-step flow of communication^47,48^ where influencers are opinion leaders who disseminate information to their followers, accelerating the process of diffusion.^49^ However, adolescents view influencers they follow as more than opinion leaders in that they perceive themselves as having an emotional connection with their preferred influencers, otherwise known parasocial relationships.^50^ As such, adolescents often perceive having meaningful ties with influencers, similar to real-life social connections they have with people close to them.^51,52^ This perceived emotional connection is strengthened further as influencers sometimes communicate directly with their followers through written comments on social media or live streaming of videos.^53^ Further, adolescents experiencing this dynamic see the influencer as a role model and often align their values with that of the influencer.^52^ Consequently, public health campaigns aimed at young people employing influencers to deliver messaging, such as tobacco uptake prevention,^54^ safe sex practices,^55^ and suicide prevention^56^ experienced much greater reach than anticipated, especially for underserved groups.^57^ Most recently, public health researchers collaborated with mental health influencers on TikTok by upskilling them with toolkits and training sessions in an attempt to address misinformation propagated by influencers, which resulted in an increase in evidence-based content and overall reach.^36^ Lastly, we conducted public involvement (PI) research in this realm, exploring adolescent girls’ (*n*  =  16) and influencers’ (*n*  = 5) views about influencer-delivered interventions to improve body image.^58^ The findings emphasised the importance of choosing an influencer whose messages and core values are aligned with the topic of body image; influencer authenticity and drawing on their personal experience; collaborating on content creation with mental health professionals; and the need for long-form (i.e. 10–20 min) content to address serious topics.^58^ The results suggested that influencer-delivered interventions could be useful for improving the body image of young people.^58^

Given the importance of learning directly from adolescents – the intended end users – about their needs and how to meet them via interventions,^59,60^ we aim to build upon previous public health and PI research^54–58^ through additional research with a larger and more representative sample of adolescent girls in the United Kingdom. This study employs a mixed-methods online survey to expand our understanding of their perspectives about influencer-delivered interventions, not only around body image, but also mental health more broadly. To meet this overall objective, this study has three research questions that serve to inform future intervention development by providing essential context around what adolescent girls think are important factors relating to influencers in general, and also in relation to body image and mental health support: (1) What do adolescent girls think are important qualities in influencers? (2) What do adolescent girls think would be useful when receiving and/or seeking out support for body image issues, and how does this relate to influencers? (3) What do adolescent girls think would be useful when receiving and/or seeking out support for mental health and wellbeing issues, and how does this relate to influencers?

## Method

### Participants and procedure

Inclusion criteria included being a UK-based adolescent girl; a user of YouTube and/or TikTok; and being between the ages of 13 and 18 given that the minimum age for most social media platforms is 13 years.^61^ As our study was exploratory (i.e. not based on hypotheses; no statistical tests were conducted), a power analysis was deemed unnecessary.^62^ Our sample size (*N*  = 375) is in line with studies employing similar designs.^63–65^

Recruitment was conducted by QRS Market Research Ltd, a UK-based research agency via their recruitment panels of participants (i.e. teens and their parents) they successfully used in the past for research with this target group. For girls between the ages of 13 and 17 years, the research agency obtained informed written consent from their parents, and girls provided informed assent at the beginning of the survey. Girls 18 years of age provided informed written consent. All participants were sent a unique link via email to access and complete the survey; parents of those under the age of 18 received the link to complete the demographic information before handing the device over to their daughter to complete the survey.

### Survey

**Figure 1.**
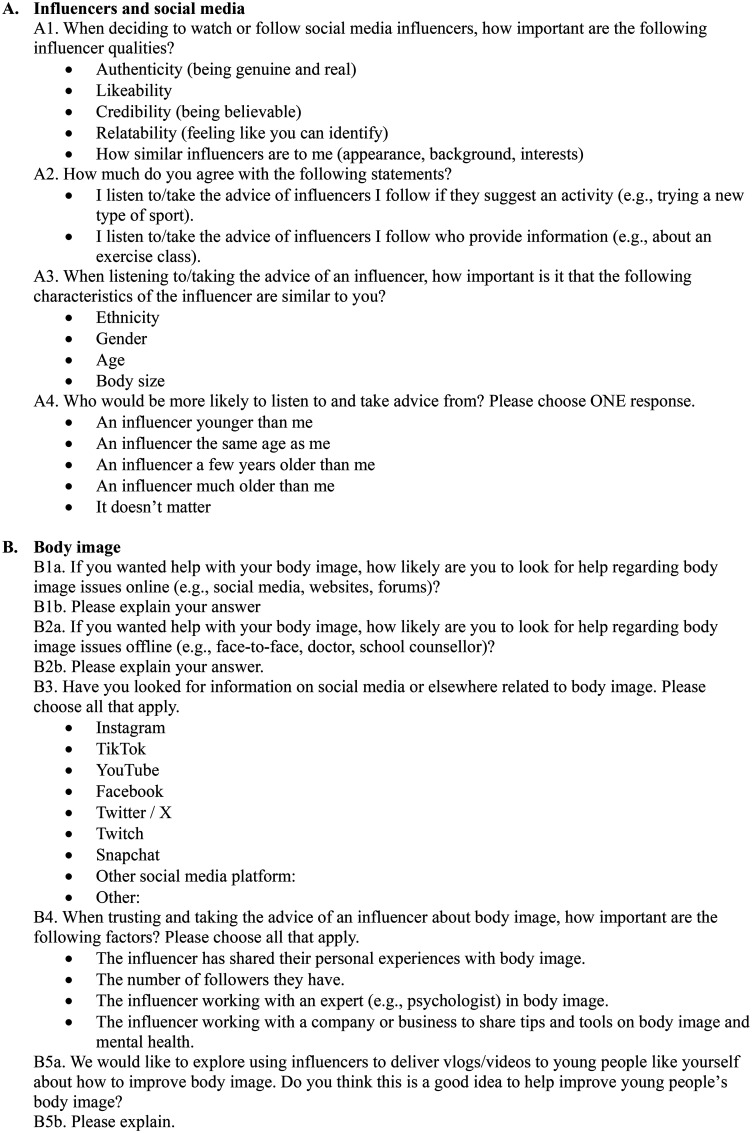

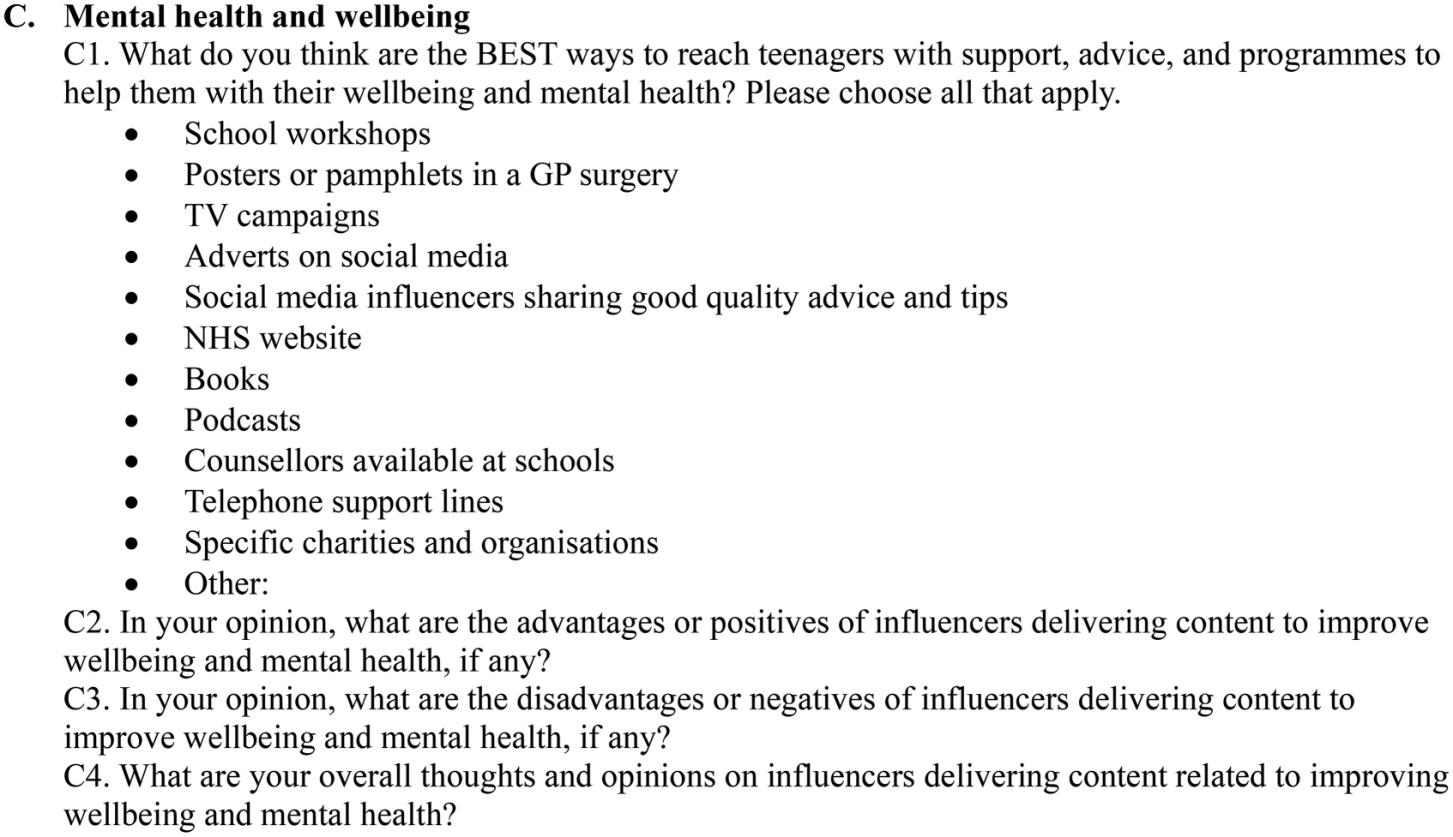
Summary of the survey structure.

To ensure the survey was user-friendly and appropriate for the target audience, the first author tested its readability using an online tool,^66^ which confirmed it would be easily understood by 13- to 14-year-olds. Additionally, adolescent girls (*n *= 5) obtained through contacts of the research team and research agency reviewed the survey, resulting in minor revisions to maximise comprehension. Following these survey revisions, the research agency uploaded the survey created by the investigators to their in-house survey platform, Forsta (2024.6.28). The investigators (SH and NP) tested the functionality of the survey before the research agency shared with participants. Following the initial demographic questions, the survey was formatted into three sections. Section A, *Influencers and social media*, primarily included questions relating to girls’ opinions on the characteristics of influencers they deem important. Section B, *Body image*, asked participants where they would seek out support for their body image if they required it, if they have sought out information on body image and from where, factors they deem important in influencers who share information on body image, and their opinions on influencers delivering body image vlogs. The last section, section C, *Mental health and wellbeing*, asked participants to share their thoughts on the best place to seek wellbeing and mental health support, as well as the advantages and disadvantages of having influencers delivering content to improve wellbeing and mental health. The survey consisted of 31 items^1^: 6 demographic questions, 15 close-ended questions with Likert-scale or multiple-choice response options, and 10 open-ended questions to provide participants an opportunity to share further details of their perspectives.^67^ Figure 1 outlines a summary of the survey structure following the demographic questions, featuring only the questions that were analysed (i.e. 10 close-ended questions and 6 open-ended questions). See Supplemental material 1 to view the full survey.

### Data analyses

The quantitative data from the multiple-choice questions were analysed through descriptive statistics using SPSS (version 29.02.0). The qualitative data from the open-ended questions were analysed through content analysis^68^ using Excel (version 16.90). Inductive (i.e. data driven) content analysis – moving from the specific to the general – was undertaken given the limited literature in this realm.^68^ Assuming a constructivist perspective, the data guided the creation of subcategories and categories (i.e. no pre-determined groupings were imposed upon the data), thus centring the participants’ perspectives. Consistent with this approach, authors SH and NP engaged in reflexivity throughout the analytic process, recognising that their perspectives as researchers with experience working with influencers and adolescent girls inevitably interacted with the data.^69^ Specifically, the authors remained conscious of ensuring all varied perspectives, regardless of whether they supported the notion of influencers delivering interventions or not, were fairly represented in the analysis. Each of the qualitative questions were analysed separately. The first author/SH led the coding process and created the preliminary subcategories per question, which were reviewed by and discussed with the senior author/NP, which resulted in collapsing similar subcategories and creating a few new subcategories. After further refinement, SH and NP finalised the subcategories for each of the qualitative questions. Next, SH evaluated the subcategories across questions (i.e. all qualitative data) and grouped together similar subcategories to create larger generic categories across the qualitative dataset. Following, SH and NP reviewed and agreed upon the generic categories together. Lastly, in line with content analysis, SH clustered the generic categories that related to each other to form the overarching main categories. All subcategories, generic categories, and main categories were reviewed once again by SH and NP before finalising. The reporting of the qualitative data adhered to the Standards for Reporting Qualitative Research guideline^70^ (see Supplemental material 2).

## Results

### Demographic characteristics

A total of 375 UK-based adolescent girls between the ages of 13 and 18 years (*M*  = 15.39, *SD*  = 1.63) completed the survey (see Table 1).

**Table 1.** Participant demographic data.

		Total (*N* = 375)
Age years, *M* (*SD*)		15.39 (1.63)
Age (years), *n* (%)		
	13	59 (15.7)
	14	65 (17.3)
	15	84 (22.4)
	16	54 (14.4)
	17	64 (17.1)
	18	49 (13.1)
Ethnicity, *n* (%)		
	Bangladeshi	5 (1.3)
	Black African	30 (8.0)
	Black British or any other Black background	6 (1.6)
	Chinese	2 (0.5)
	Indian	6 (1.6)
	Mixed origin/multiple ethnic background – White and Black Caribbean	4 (1.1)
	Mixed White and Asian	8 (2.1)
	Mixed White and Black African	4 (1.1)
	Other Asian background	2 (0.5)
	Other mixed/multiple ethnic background	3 (0.8)
	Other White background (e.g. European, American, etc.)	5 (1.3)
	Pakistani	11 (2.9)
	White English / Welsch / Scottish / Northern Irish / British	277 (73.9)
	White Irish	6 (1.6)
	Any other ethnic group*	5 (1.3)
	Prefer not to say	1 (0.3)
Region in the UK, *n* (%)		
	Greater London	48 (12.8)
	South East	43 (11.5)
	South West	34 (9.1)
	West Midlands	38 (10.1)
	North West	36 (9.6)
	North East	25 (6.7)
	Yorkshire and Humberside	32 (8.5)
	East Midlands	26 (6.9)
	East Anglia	26 (6.9)
	Wales	24 (6.4)
	Scotland	31 (8.3)
	Northern Ireland	12 (3.2)
Socioeconomic status**, *n* (%)		
	A/B (Higher and intermediate managerial, administrative, and professional occupations)	110 (29.3)
	C1/C2 (Supervisory, clerical, and junior managerial, administrative and professional occupations; skilled manual occupations)	203 (54.1)
	D/E (Semi-skilled and unskilled manual occupations; unemployed and lowest grade occupations)	62 (16.5)
Number of hours per week on YouTube, *n* (%)		
	Less than 1 hour	35 (9.3)
	1–2 hours	65 (17.3)
	2–4 hours	73 (19.5)
	4–6 hours	73 (19.5)
	6–8 hours	51 (13.6)
	8–10 hours	37 (9.9)
	More than 10 hours	41 (10.9)
Number of hours per week on TikTok, *n* (%)		
	Less than 1 hour	54 (14.4)
	1–2 hours	51 (13.6)
	2–4 hours	51 (13.6)
	4–6 hours	55 (14.7)
	6–8 hours	56 (14.9)
	8–10 hours	39 (10.4)
	More than 10 hours	69 (18.4)

*Other ethnicities included Brazilian, European, Macedonian, Ukrainian, White Latina.

**Based on the household's chief income earner. Categories of Social Grade as per the Office for UK National Statistics.^71^

### Quantitative analysis

**Figure 2.**
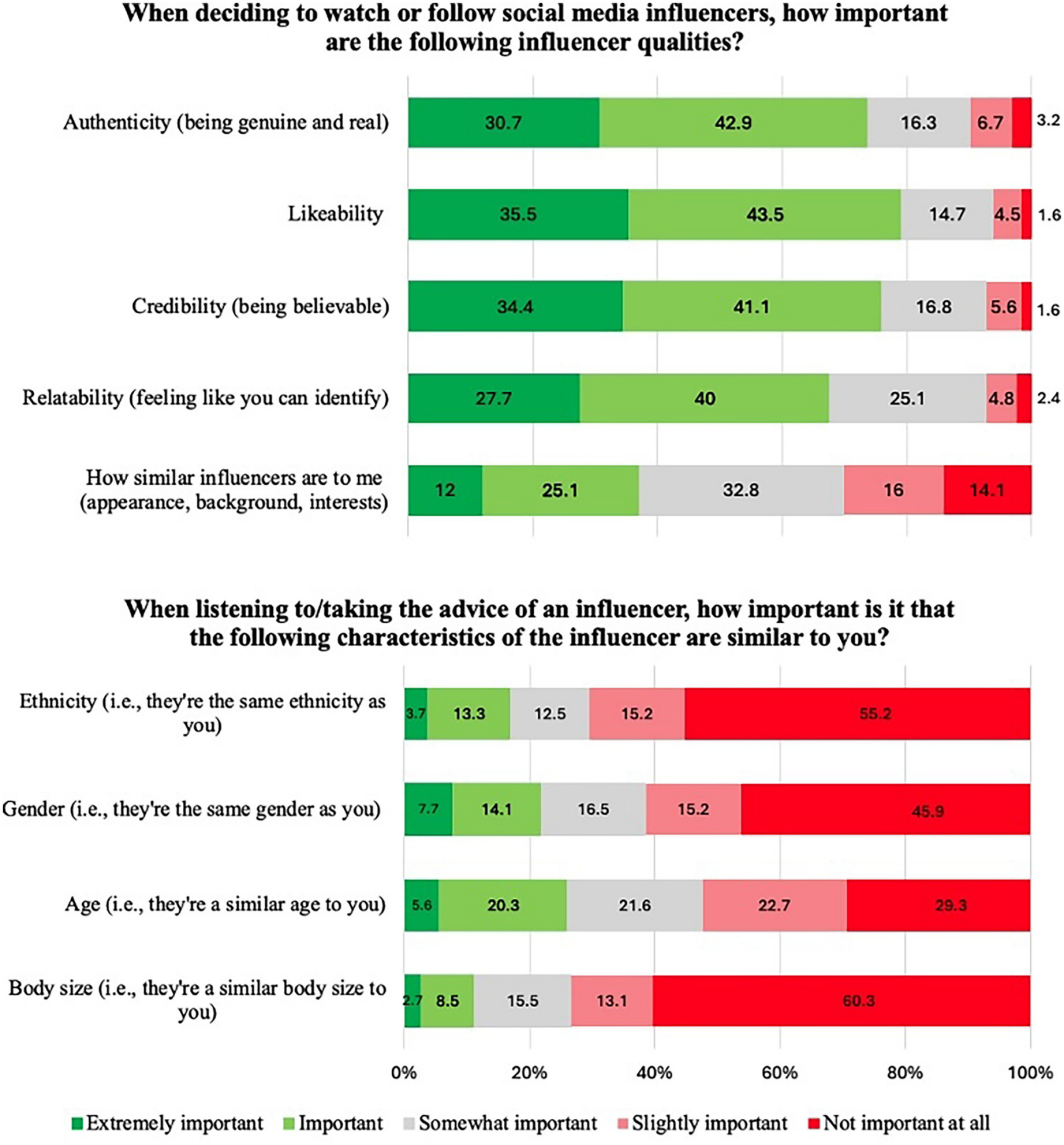
Distribution of responses to questions A1 and A3 relating to influencer qualities and characteristics. Responses are ordered from ‘extremely important’ to ‘not important at all’.

In section A (*Influencers and social media*), questions A1, A3, and A4 inquired about the qualities and characteristics of influencers that girls deemed important. Specifically, when deciding to watch or follow an influencer, the majority of participants (between 67% and 79%) felt it was extremely important or important that influencers were likeable (79% vs. 6% who indicated it was not important at all and slightly important), credible (76% vs. 7%), authentic (74% vs. 10%), and relatable (68% vs. 7%) (see Figure 2). When listening to or taking the advice of an influencer, more than half of participants did not think it was important (i.e. not important at all and slightly important) that the influencer was the same ethnicity (70% vs. 17% who indicated it was extremely important and important), gender (61% vs. 22%), or body size (73% vs. 11%) as themselves. Although the responses to question A3 regarding age were mixed (see Figure 2), the responses to question A4 found that almost half (48%) said that they would be more likely to listen to and take advice from an influencer a few years older than themselves, whilst 31% said it didn’t matter, 14% reported they would prefer an influencer the same age as themselves, 7% preferred an influencer much older than themselves, and less than 1% said they would prefer a younger influencer. The responses to question A2 revealed that more than 40% of participants mostly/totally agreed that they would listen to and take the advice of influencers if the influencer suggests an activity (46% vs. 18% who mostly/totally disagreed) or provides information (56% vs. 14%).

**Figure 3.**
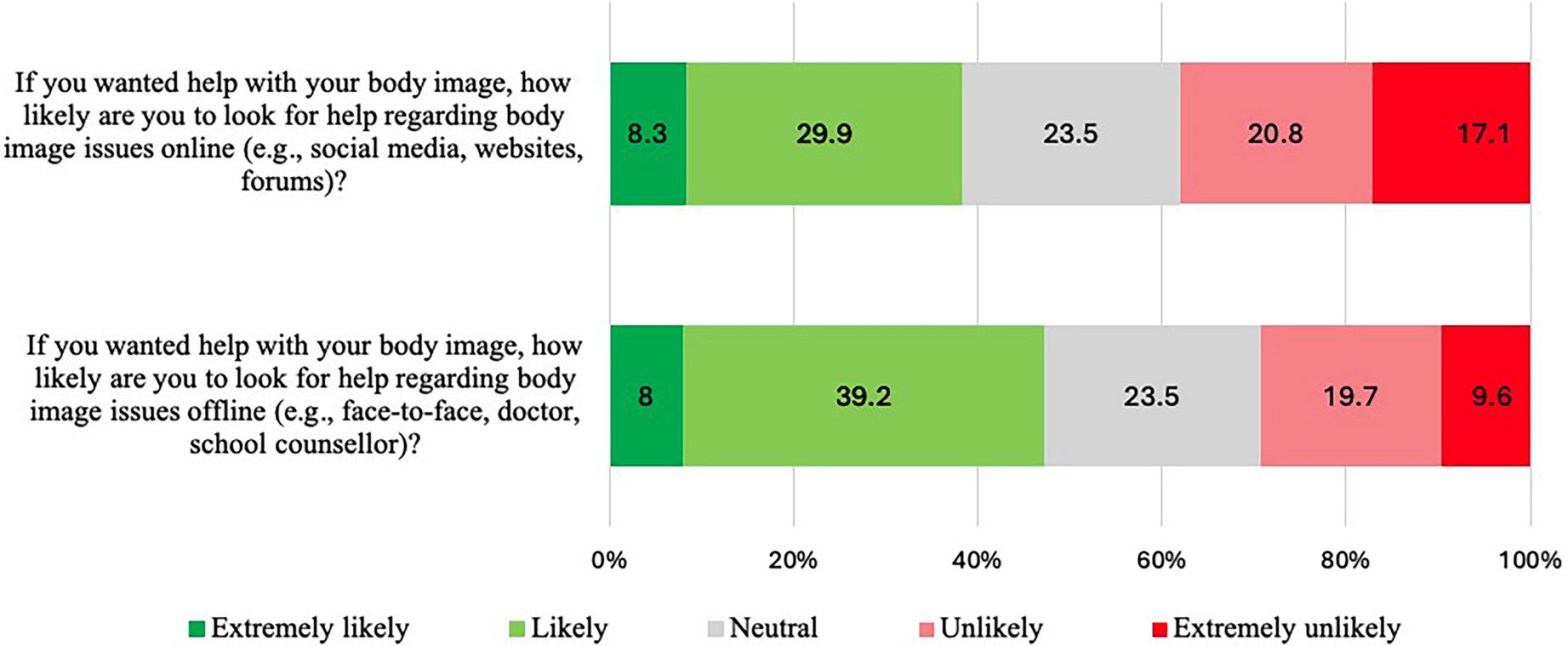
Distribution of responses to questions B1a and B2a. Responses are ordered from ‘extremely likely’ to ‘extremely unlikely’.

Five close-ended questions in section B (*Body image*) were analysed. Questions B1a and B2a explored the likelihood of participants seeking body image support online and offline. Results showed that slightly more participants were extremely likely or likely to seek out support offline (47%) as compared to seeking out support online (38%) (see Figure 3).

**Figure 4.**
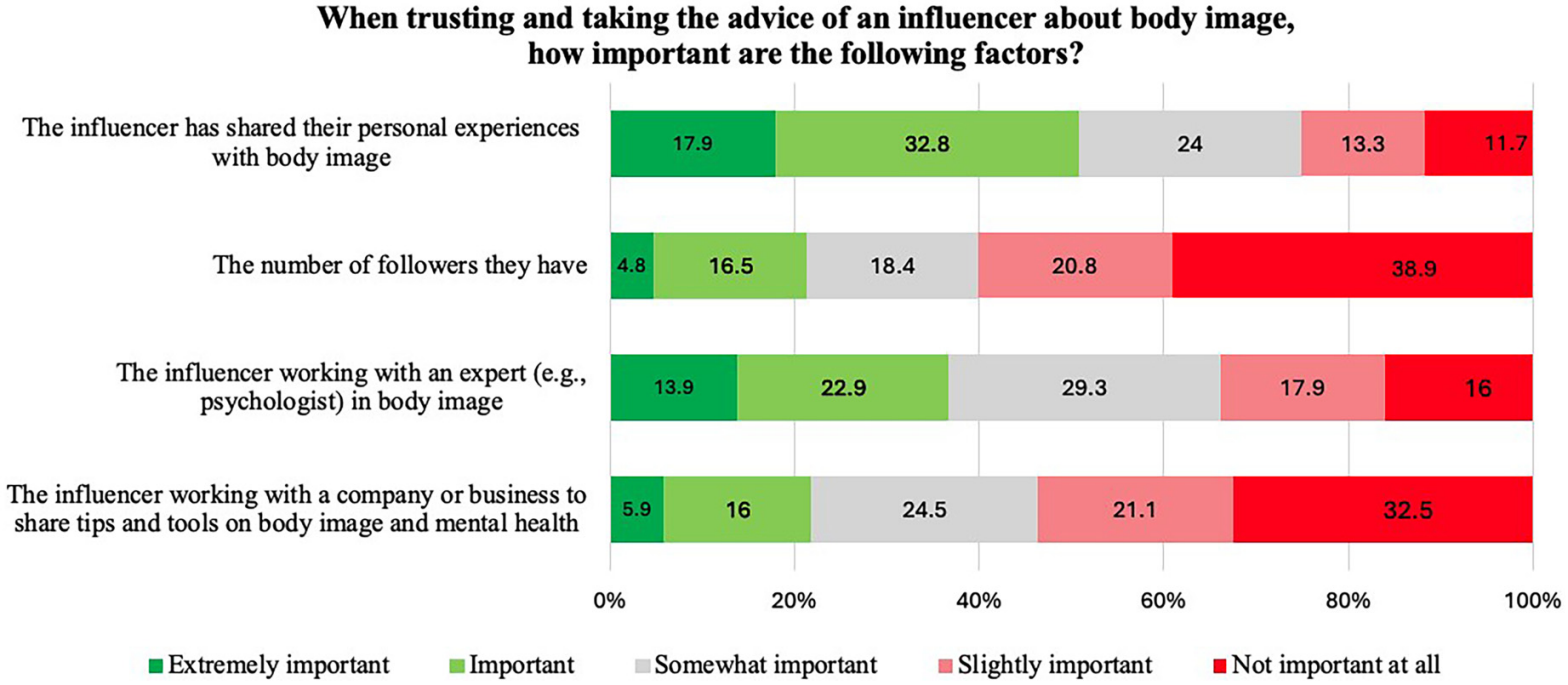
Distribution of responses to question B4. Responses are ordered from ‘extremely important’ to ‘not important at all'.

Responses to question B3 revealed that the top three online sources that young people have turned to for body image information were TikTok (46%), YouTube (38%), and Instagram (30%). Other online sources participants accessed for the same purpose were comparatively much lower, which included Facebook (14%), Snapchat (11%), Twitter/X (6%), Twitch (2%), Pinterest (1%), Google (0.3%) and the National Health Service (0.3%). Results regarding what participants thought were important factors when trusting and taking the advice of an influencer about body image (question B4) revealed the most important factor (i.e. extremely important and important) was that the influencer has shared their personal experiences with body image (51%) as opposed to 25% who deemed it not important at all and slightly important. The second most important factor was that the influencer works with a body image expert (e.g. psychologist) (37%); however, results were mixed as 34% of participants deemed this as ‘not important at all’ and ‘slightly important’ (see Figure 4).

With regard to question B5a, most participants totally agreed (20.3%) or mostly agreed (41.1%) that using influencers to deliver vlogs/videos to young people to improve their body image was a good idea; 25.3% neither agreed nor disagreed, 8.5% mostly disagreed, whilst 4.8% totally disagreed.

The last section (*Mental health and wellbeing*) included one close-ended question (C1), which asked participants what they thought were the best ways to reach adolescents with support advice and programmes to help them with their wellbeing and mental health. Results indicated that social media and school environments were their top preferences: influencers sharing good-quality advice and tips (52%), school workshops (51%), counsellors available at schools (45%), adverts on social media (41%), NHS website (33%), podcasts (27%), telephone support lines (25%), TV campaigns (23%), books (16%), specific charities and organisations (15%), and posters or pamphlets in a GP surgery (13%).

### Qualitative analysis

Inductive content analysis was conducted on questions B1b, B2b, B5b, C2, C3, and C4. See Supplemental materials 3 to 6 for the subcategories and example quotes per question. Whilst most of participants’ responses per question were coded once, responses that included two separate ideas were double-coded (i.e. coded to two different subcategories), so as to preserve the richness of the data.^72^ The response rate per question ranged between 92% and 94%, and a total of 1392 comments were analysed. Three main categories were identified, each containing two to three generic categories as derived from the subcategories.^68^ Table 2 presents the complete results of the content analysis (i.e. subcategories, generic categories, and main categories).

**Table 2.** Content analysis results of the open-ended responses within the survey outlining the subcategories, generic categories, and main categories of survey participants’ open-ended responses (*N* = 1392).

Main categories	Frequency *n* (%)	Generic categories	Frequency *n* (%)	Subcategories*	Frequency *n* (%)	Example quotes
Influencer-delivered interventions would be helpful	745 (53.5)	Can offer help / support	529 (71.0)	Can obtain general advice and support	310 (58.6)	‘I would look for people who relate to what I'm feeling, then try to find advice on how to deal with it.’
				Helpful for health and wellbeing	52 (9.8)	‘for health reasons’
				Body image is an important issue	29 (5.4)	‘Body image is such a leading source of mental health problems for young women’
				Can learn from others with same issue	28 (5.2)	‘I would like to get suggestions online from people who has [sic] experienced what I try to do’
				Could be useful in conjunction with experts	26 (4.9)	‘I think if an influencer has the right information from professionals and has experience then it could help many people’
				General positive comments	26 (4.9)	‘This is important’
				Could be useful if ‘done right’ (non-specified)	19 (3.6)	‘If done well can be a great help’
				Use to learn about exercise and diets	18 (3.4)	‘Great work out [sic] routines on youtube’
				Raise awareness	12 (2.2)	‘Speaking about it makes more people aware of it’
				Reduce mental health stigma	5 (1.0)	‘to overcome any barriers about the issue’
				Could be useful in conjunction with offline support	4 (1.0)	‘I am supportive of this, but perhaps more offline content should also be considered such as in person visits by influencer or school workshops’
		Vlogs delivered by influencers online is an ideal forum	202 (27.1)	Provides anonymity	90 (44.6)	‘being online so you stay unknown’
				Huge reach	38 (18.8)	‘They have a wide network of followers’
				Easy access	51 (25.2)	‘It's convenient’
				The internet is a good vehicle to reach young people	23 (11.4)	‘I feel like this is a good place to start with as most young people nowadays are on social media, so would be more likely to see it.’
		Can increase confidence and skills in viewers	14 (1.9)	Can increase confidence	8 (57.1)	‘It’ll be helpful for young people to gain confidence in themselves’
				Can increase media literacy skills	3 (21.4)	‘because everyone looks perfect behind a screen but they aren't no one is perfect’
				Vlogs would be interesting and inspiring	3 (21.4)	‘It inspires us’
Influencer-delivered interventions would be unhelpful	391 (28.1)	Influencers are not qualified	179 (45.8)	Could receive incorrect information	103 (57.5)	‘biased opinions and unhealthy opinions’
				Could cause harm	47 (26.3)	‘I think wrong information could easily influence people in a bad way’
				General negative comments	22 (12.3)	‘Not a good idea’
				Lack appropriate training	7 (3.9)	‘Not their place as everyone is different and these people will not be trained’
		In-person support would be more helpful	168 (42.9)	Prefer to see a professional	69 (41)	‘I trust my GP to give me the best advice possible’
				Prefer family and friends	50 (29.8)	‘I would talk to my mom or friends or brother’
				More appropriate / easier	25 (14.9)	‘might be easier’
				Personalised help	17 (10.1)	‘because generic advice is being given to people they know nothing about’
				Influencer content will only reach their followers	7 (4.2)	‘Not all people will see it’
		The internet is a toxic environment	44 (11.3)	Unrealistic beauty standards online	29 (66.0)	‘People could be using filters’
				Negative commentary by others online	7 (16.0)	‘Too many people with nasty comments’
				Influencers can trigger comparisons	4 (9.0)	‘Can make people feel like they are always comparing their self to others’
				Influencer could have bad reputation or be ‘cancelled’	4 (9.0)	‘Well, when these influencers have a [sic] bad reputations, they might cause complicated feelings’
Influencer qualities	255 (18.3)	Positive qualities	161 (63.1)	Relatable	48 (29.8)	‘Relatable – already known to people – more likely to be receptive to messages’
				Can relate to mental health issues	32 (19.9)	‘Tell their own stories and how they coped’
				Young people will listen	30 (18.6)	‘Cos [sic] teenager [sic] pay attention to them. They’re more likely to get through to people like me than other grown ups’
				Trustworthy	19 (11.8)	‘A lot of kids trust influencers’
				Can relate to body image issues	8 (5.0)	‘I think that positive social media content could help dilute the toxic and potent beauty standards perpetuated by influencers who are also under the high pressures to conform to the beauty standard’
				Inspirational	16 (9.9)	‘They are people most teenagers look up to and follow’
				Genuine	8 (5.0)	‘It's good when people I follow discuss things that are going on in their own life’
		Negative qualities	94 (36.9)	Untrustworthy	62 (66)	‘I don’t view influencers as the most reputable or trustworthy people on topics like body image or mental health’
				Can promote appearance ideals	22 (23.4)	‘They have unrealistic bodies’
				Influencers are motivated by money	5 (5.3)	‘Useless money grabbing’
				Unrelatable	5 (5.3)	‘might feel like they don't relate to having bad mental health’

*Open-ended responses relating to Likert-scale neutral responses, subcategories of ‘miscellaneous’, ‘don’t know/not sure’, ‘don’t need help’, and ‘not interested’ were not included in the generation of the generic and main categories.

The largest main category, *Influencer-delivered interventions would be helpful*, constituted just over half of all responses, and included three generic categories: *Can offer help and support*; *Vlogs delivered by influencers online is an ideal forum*; and *Can increase the confidence and skills of viewers*. The first generic category encompassed over 70% of the responses; its most populated subcategories referred to general advice and support, *helpful for health and wellbeing*, and *body image as an important issue*. The second generic category – *Vlogs delivered by influencers as an ideal forum* – had four subcategories (i.e. *provides anonymity*, *huge reach*, *easy access,* and *young people spend a lot of time online*), with *provides anonymity* being the largest subcategory. The final generic category, *Can increase viewers’ confidence and skills*, constituted the least number of responses.

The second largest main category identified – *Influencer-delivered interventions would be unhelpful* – consisted of just over a quarter of all responses and three generic categories. The first generic category, *Influencers are not qualified*, was composed of four subcategories. The first two subcategories constituted most of the responses in this generic category: *could receive incorrect information* and *could cause harm*. The second generic category, *In-person support would be more helpful*, was comprised of five subcategories, with the first two subcategories occupying most responses: *prefer to see a professional* and *prefer family and friends*. The final generic category was *The internet is a toxic environment*, and its largest subcategory was *unrealistic beauty standards*, constituting two thirds of this generic category.

The final and smallest main category, *Influencer qualities*, was split into the generic categories of *Positive qualities* and *Negative qualities*, with the former constituting two-thirds of responses. The largest subcategories for *Positive qualities* were *relatabl*e; *influencers can relate to mental health issues*; and *young people will listen to influencers*. The top subcategories for *Negative qualities* were *untrustworthy* and *can promote appearance ideals*.

## Discussion

### Main findings

The overall aim of this study was to understand adolescent girls’ perspectives around influencer-delivered interventions to improve body image, and mental health more broadly, building on and expanding previous research with UK-based adolescent girls.^58^ To gain a nuanced understanding of these views, our mixed-methods online survey aimed to answer three research questions, which are discussed below.

The first research question explored what adolescent girls viewed as important qualities in influencers to help inform future intervention development. Quantitatively, the majority of the sample agreed that likeability, credibility, authenticity, and relatability were all key qualities that helped determine whether to consume content by an influencer. Qualitatively, the main category of *Influencer qualities* revealed two-thirds of girls thought that overall, influencers possess more positive qualities than negative ones, with the most salient positive quality being relatability. Taken together, these results align with previous research in that perceived likeability,^73^ credibility,^74^ relatability,^75^ and authenticity^76^ have all been deemed as significant factors by young people who follow influencers, which contribute to the development of a perceived intimate connection with the influencer (i.e. a parasocial relationship).

In looking at the negative qualities of influencers that girls provided in their open-ended responses, the standout quality was untrustworthiness. Underscoring the importance of this finding is that perceived trustworthiness has been found to be more influential than the information presented in the content^77^ or an influencer's expertise when it comes to change.^78^ In part, this could be attributed to the huge number of influencers to choose from and the inevitable variation of the content they share, coupled with individual differences and preferences of their followers. However, parasocial relationships play a critical role in the perceived trustworthiness of an influencer, helping to explain why some young people follow the advice of specific influencers but not others. Chung and Cho^79^ found strong perceived bonds are essential in generating trust among an influencer's followers. Specifically, using mock advertisements featuring various celebrities, they found support for their proposed model whereby young people's previous social media interactions with the featured celebrities, particularly those who engaged in self-disclosure, revealed stronger parasocial relationships than with celebrities they hadn’t previously engaged with, which led to higher levels of trust, and ultimately increased purchase intentions.^79^ Additionally, Kim and Kim^80^ examined the role of trust in influencer marketing, where participants identified an influencer whose content they frequently engaged with over the last month and then rated the influencer on various characteristics. Results revealed that influencers’ perceived authenticity, expertise, and similarity in values and experiences to the user were all key in being trusted by their followers.^80^ Further, De Jans et al.^81^ reinforces the necessity of credibility in building trust through their examination of adolescents’ perceptions of influencers who include advertising disclosure statements on their paid content. They found such disclosures reduced young people's trust in the influencer, as they perceived the influencer to be sharing a biased opinion and being manipulative with their viewers.^81^ As such, young people's trust in influencers can be seen as a product of how authentic, credible, and relatable they perceive them to be. Recognising the importance of trust and the main qualities that contribute to building trust is key when considering the selection of an influencer to deliver mental health interventions to adolescent girls.

We also explored the importance of how similar influencers are to their followers (i.e. appearance, background, and interests) when listening to or taking the advice of an influencer to provide guidance in selecting potential influencers to deliver interventions to adolescent girls. These quantitative findings produced mixed results. Although slightly more participants identified that they agreed it was important (37.1%), there were roughly equal numbers of participants who agreed, disagreed (30.1%), or neither agreed nor disagreed (32.8%). As noted, one of the key qualities Kim and Kim^80^ identify as contributing to building trust in an influencer is the perceived similarity of values and experiences. Related evidence about the role of homophily – the tendency of people to be drawn to and interact with people who are objectively or subjectively similar^82^ – suggests that the perceived similarity of influencers by users encompasses different dimensions. Specifically, Ladhari et al.^83^ examined the four dimensions of homophily as originally conceptualised, namely shared attitudes, values, appearance, and background.^84^ They found that, in order of importance, attitudes, values, and appearance were the key dimensions women users considered when defining an influencer as similar to themselves.^83^ This evidence suggests that had we defined similarity separately on dimensions of attitudes, values, and appearance for participants, the findings might have been clearer in terms of how important perceived similarity with an influencer is for them. As such, future research could explore the dimensions of homophily with adolescent samples to provide clarity.

That said, we quantitatively explored the dimension of appearance relating to body size, ethnicity, gender, and age. Almost three-quarters of participants indicated that body size (73.4%) and ethnicity (70.4%) were not important, in addition to 61.1% and 52% of participants with regard to gender and age, respectively. These results could be partially explained given that the dimension of appearance is the least important dimension of homophily.^83^ Current related literature is lacking, as only a few studies involving adult participants examine the relationship between appearance-based characteristics and the strength of the parasocial relationship among influencers and their followers.^85–87^ There is a clear need for additional evidence, particularly surrounding adolescents’ perspectives about the importance of sharing the same gender, ethnicity, and body size as an influencer, which underscores the novelty of our findings.

With regard to age, almost half of participants indicated they would be more likely to listen to and take the advice of an influencer a few years older than themselves, which coincides with body image literature around peer-led interventions.^88^ Taken together, our results suggest that appearance-based characteristics are not central when seeking an influencer to deliver mental health interventions. However, there is a caveat. The second-most popular subcategory of the *Negative qualities* generic category was that influencers can promote appearance ideals, either through their own appearance (e.g. ‘usually really skinny and pretty’) or content (e.g. ‘spread false information about the way the body looks’). Along similar lines, participants who didn’t feel influencer-delivered interventions would be helpful also expressed that the internet can be a toxic environment, citing unrealistic beauty ideals as the top reason. Therefore, when seeking out influencers to deliver a mental health intervention with at least one body image outcome, influencers who value appearance diversity should be prioritised.

In addition to the influencer characteristics we enquired about, which were salient in our previous study^58^ and reflect the overall evidence base,^73–81^ further exploration of other influencer characteristics that girls may deem important is warranted. For instance, influencers with a sense of humour can strengthen an influencer's ability to persuade,^89^ and influencers who share the same location as their followers can increase followers’ social identification with them.^90^ Relatedly, our study did not explore the intersection of influencer characteristics, which could provide further understanding around the appeal of influencers among adolescent girls. For instance, Tafesse and Wood^91^ explored Instagram content and engagement strategies of more than 200 Instagram influencers. They found that influencers with a high follower count had lower engagement when they posted about varied topics, but influencers with various interests saw greater engagement when they posted more often,^91^ suggesting future research around the interaction between influencer characteristics on YouTube and TikTok could shed light on nuances specific to influencers popular with adolescent girls.

Building on the two-step flow of communication model,^47,48^ Uses and Gratifications Theory (UGT) led by the same theorist^92^ can provide further context as to why adolescents trust and engage with influencers and the content they share. UGT centres around the concept that people are active agents who aim to have specific needs or gratifications met through what they consciously choose to consume in the media, namely, entertainment, personal identity, information, and social interaction.^93^ Parasocial relationships can act as one mechanism for having one or more of these needs met.^94,95^ Whilst existing literature is limited, the evidence indicates that adolescents and young adults are motivated to follow and engage with influencers to fulfil their needs around information-seeking and entertainment,^96,97^ as well as socialisation.^98^ Additional evidence suggests that the need of identity construction can be facilitated by sharing content created by influencers.^96,99^ Although our study did not aim to understand adolescents’ specific motivations for following influencers, our findings suggest that information-seeking (i.e. influencer-led interventions can provide helpful advice) is one reason adolescent girls may consume influencer content. Future research around why adolescent girls follow influencers in the context of UGT can offer insights to add to our current understandings, with the caveat that social media platforms may differ in the gratifications sought.^100^

Due to the overlap in participants’ responses, and that body image is a mental health issue, our analysis of the open-text content was undertaken as a whole. As such, the second and third research questions – exploring what adolescent girls thought would be useful when receiving and/or seeking out support for issues with body image (second research question) and mental health more broadly (third research question) in relation to influencers – are discussed here in tandem. Given our interest in delivering interventions via influencers, a key quantitative question explored the likelihood of girls seeking out body image support online. Findings indicated that somewhat more participants were likely to look for support offline (47%) as opposed to online (38%). However, our content analysis results revealed that the largest main category was centred around girls asserting that influencer-delivered interventions would be helpful (53.5%). In breaking down why, a key reason is that girls viewed the internet as an ideal forum, citing anonymity as the most important reason, reinforcing previous research.^26^ The qualitative data related to their desire for anonymity and subsequent preference for online support elucidated that feelings of embarrassment were central, such as, ‘I would be too embarrassed to talk face to face’ and ‘I might feel embarrassed talking to someone I know’. This provides helpful context in understanding the participants who indicated they had already actively sought out body image-related information on social media, primarily via TikTok, YouTube, and Instagram. As embarrassment is linked closely with mental health stigma,^26^ this key barrier could potentially be overcome by having influencers deliver evidence-based support. Similarly, girls thought the best ways to reach adolescents with mental health support also identified social media (i.e. influencers and advertisements) as a key information-delivery mechanism. Notably, equally popular responses identified schools as an ideal environment for mental health support, through workshops and on-site counsellors. Both preferences for obtaining mental health support (i.e. social media and schools) reflect where adolescents spend much of their time and highlight the importance of disseminating interventions to young people where they’re at.

Naturally, individual preferences play a role in help-seeking. For instance, the 28% that expressed influencer-delivered interventions would be an unhelpful approach highlighted that in-person support would be their first port of call if needed, primarily through professionals, followed by family and friends. Some girls’ preference for professional mental health support contextualises the most popular reason they believe that influencer-delivered interventions would not be helpful: influencers are not qualified. Their most notable concerns are that influencers could spread misinformation and cause harm, reflecting a systematic review by Freeman et al.,^101^ which found that adolescents experienced a general distrust of health information disseminated on social media. Accordingly, the potential for harm through unqualified influencer content must be carefully considered in the context of intervention development. Future efforts should explore regulatory frameworks and co-design with clinical experts to ensure ethical implementation. Relatedly, our quantitative results showed just over a third of girls thought it was important influencers collaborate with an expert if delivering a body image intervention, which could circumvent their valid concerns of disseminating incorrect or harmful content. That said, roughly equal numbers of girls took a neutral stance or didn’t think that influencer-expert collaboration was important. Although there is limited evidence around the impact of credible influencer-delivered content, TikTok mental health influencers who attended evidence-based training and were provided with toolkits to assist with content creation based on science saw their content obtain hundreds of thousands additional views.^36^ As such, although the majority of our sample did not prioritise influencers working with experts, this evidence suggests that doing so could potentially not only increase the amount of scientifically sound content on social media, but also its uptake due to increased credibility.

Interestingly, just over half indicated that it was important for the influencer to share their personal experiences with body image, which could lead young people to view them as experts by experience. Indeed, some influencers open up to their followers about their mental health issues,^46^ which has been shown to be a salient predictor of parasocial relationships.^79^ For instance, in the aforementioned research with TikTok mental health influencers, more than half of the 11 influencers that participated in qualitative interviews created content based on their lived experience.^102^ Relevant self-disclosure was reflected in the positive qualities of influencers identified by participants; namely, they valued that some influencers can personally relate to mental health and body image issues. Such concerns are deeply personal, sensitive topics. As such, when choosing influencers to deliver mental health interventions, previous experience in disclosing their own mental health struggles may indicate an especially loyal following, regardless of its size. For instance, Rasmussen et al.^103^ found that even influencers with 100,000 followers can develop strong bonds with their followers. Similarly, influencer-delivered intervention content must be authentic, as emphasised in previous research with adolescent girls and influencers who expressed content must reflect the influencer's lived experience.^58^ Accordingly, special consideration should be given to those influencers who self-disclose around issues related to the intervention with the understanding that it strengthens trust in their followers.

Academics collaborating with influencers to deliver mental health interventions to adolescents should be cognizant of the content influencers wish to share in this context, particularly around their own mental health concerns, to mitigate potential distress in the user. One such mechanism is the use of trigger or content warnings, which are statement labels identifying that the content may be upsetting to the viewer.^104^ Unfortunately, the current evidence base has not yet explored trigger warnings among adolescents, and evidence is limited around such warnings on social media.^105^ However, a qualitative study among young adults revealed participants preferred trigger warnings on sensitive social media content that identified the topic in question to help them make an informed decision as whether to engage with the content or not.^105^ Along these lines, social media platforms encourage the inclusion of content warnings in relation to sensitive mental health content, such as self-harm and eating disorders, in addition to prohibiting any content that promotes harm or presents a significant risk to viewers.^106,107^ Further, they also provide content creators (i.e. influencers) with best practices when posting about such content.^106^ Additional safeguards for users via signposting of relevant resources, such as helplines and mental health support organisations, should be included in the vlog itself and in the text beneath the vlog. Lastly, to protect young users from cyberbullying when engaging with influencer-delivered mental health interventions, influencers can block offensive users or disable the comment function.^58^

### Strengths and limitations

A central strength of this work is our commitment to centring the voices of adolescent girls in research intended to inform intervention development, as recommended by best practices,^108^ particularly for mental health research.^109^ Another strength is that our sample is much larger and more representative than our initial study with adolescent girls and influencers.^58^ Our sample's distribution across the regions and socioeconomic status throughout the United Kingdom generally corresponds to the most recent census data.^110^ Importantly, our sample, overall, has a fair proportion of minoritised ethnic groups in that almost 10% consisted of Black participants, 5.2% were from Mixed ethnic backgrounds, and almost 7% self-identified as Asian. When compared to the latest census data, our sample slightly over-represents Black groups (4.0%) and mixed ethnic groups (2.9%) but slightly under-represents Asian groups (9.3%).^111^ Whilst not a perfect reflection of ethnicities, our data includes much needed insights from demographics that are typically underserved. Although our sample was diverse, this study was not designed to assess subgroup differences in perceptions (e.g. by ethnicity or prior mental health history), which could be an important avenue for future work. One further strength was the employment of mixed methods, which provided a more in-depth examination of girls’ perspectives as opposed to taking a purely quantitative or qualitative approach. Lastly, this study adds to the nascent evidence base exploring the impact of influencers in the context of public health.^57^

This study has several limitations. One, some responses, particularly in the final sections of the survey could have been influenced by priming. Specifically, in the last section about mental health and wellbeing, we provided participants with 11 closed-ended response options for what they think would be the best ways to reach adolescents with mental health support, the final closed-text question asked of participants. The top response was ‘social media influencers sharing good quality advice and tips’, which could have been a product of priming due to the previous questions they answered about influencers. Future research could delve deeper into this finding to help understand why girls think this may be the best way to reach adolescents with mental health support. Two, social desirability may have played a role in how some girls responded. Although participants were aware that their responses were completely anonymous, and the survey was self-administered – two factors understood to reduce social desirability^112^ – it may have still contributed to the positive responses received. A third limitation is that we did not assess participants’ digital or media literacy, which may influence how adolescents evaluate the credibility of influencer-delivered content. Therefore, further research around adolescents and their individual levels of digital and/or media literacy could elucidate key differences in their perceived trustworthiness of influencers. Lastly, as evidence has established that poor body image^9^ and many other mental health concerns, such as depression,^113^ anxiety,^114^ and eating disorders^115^ disproportionately impact girls as compared to boys, our study focused only on girls’ perspectives. That said, this does not discount the existence of body image and mental health issues among boys^116,117^ and other genders.^118,119^ As such, future research should venture to explore the use of influencers among these demographics.

## Conclusion

This mixed-methods online survey study builds upon a limited but growing evidence base by providing additional insights from 375 adolescent girls in the United Kingdom about their perspectives around influencer-delivered body image and mental health interventions. Overall, the results indicate that they think that an influencer-driven approach could be helpful in improving their body image and mental health, similar to a recent qualitative interview study with adolescents and young adults, which revealed that influencers were seen as viable approach to delivering mental health support.^120^ However, the findings also revealed variation and contrasting opinions, emphasising that individual differences and personal preferences naturally play a role in the kind of influencer-created content young people want to consume.^58^ Differences also emerged with where adolescents seek out body image and mental health support. Although some preferred offline support, for the large proportion who seek out help online, influencers could be a helpful option. A key takeaway was the importance of trusting the influencer, which could be bolstered by collaboration with an expert. Additionally, other consequential influencer qualities encompassed being relatable and authentic, which could include having the influencer relate personally to the mental health issue in focus, and thus, be inclined to share their own experiences. Being similar in appearance to the influencer was not key for adolescent girls; however, they were clear in expressing that influencers who meet and/or promote beauty ideals would not be well-suited to deliver body image interventions. Taken together, the anonymity afforded by the internet and the strong parasocial bonds adolescents form with influencers support the development and evaluation of influencer-delivered interventions.

## Supplemental Material

sj-docx-1-dhj-10.1177_20552076251361340 - Supplemental material for Understanding adolescent girls’ thoughts and opinions on having social media influencers deliver body image and mental health support: A mixed-methods study Supplemental material, sj-docx-1-dhj-10.1177_20552076251361340 for Understanding adolescent girls’ thoughts and opinions on having social media influencers deliver body image and mental health support: A mixed-methods study by Sharon Haywood, Phillippa C Diedrichs and Nicole Paraskeva in DIGITAL HEALTH

sj-doc-2-dhj-10.1177_20552076251361340 - Supplemental material for Understanding adolescent girls’ thoughts and opinions on having social media influencers deliver body image and mental health support: A mixed-methods study Supplemental material, sj-doc-2-dhj-10.1177_20552076251361340 for Understanding adolescent girls’ thoughts and opinions on having social media influencers deliver body image and mental health support: A mixed-methods study by Sharon Haywood, Phillippa C Diedrichs and Nicole Paraskeva in DIGITAL HEALTH

sj-docx-3-dhj-10.1177_20552076251361340 - Supplemental material for Understanding adolescent girls’ thoughts and opinions on having social media influencers deliver body image and mental health support: A mixed-methods study Supplemental material, sj-docx-3-dhj-10.1177_20552076251361340 for Understanding adolescent girls’ thoughts and opinions on having social media influencers deliver body image and mental health support: A mixed-methods study by Sharon Haywood, Phillippa C Diedrichs and Nicole Paraskeva in DIGITAL HEALTH

sj-docx-4-dhj-10.1177_20552076251361340 - Supplemental material for Understanding adolescent girls’ thoughts and opinions on having social media influencers deliver body image and mental health support: A mixed-methods study Supplemental material, sj-docx-4-dhj-10.1177_20552076251361340 for Understanding adolescent girls’ thoughts and opinions on having social media influencers deliver body image and mental health support: A mixed-methods study by Sharon Haywood, Phillippa C Diedrichs and Nicole Paraskeva in DIGITAL HEALTH

sj-docx-5-dhj-10.1177_20552076251361340 - Supplemental material for Understanding adolescent girls’ thoughts and opinions on having social media influencers deliver body image and mental health support: A mixed-methods study Supplemental material, sj-docx-5-dhj-10.1177_20552076251361340 for Understanding adolescent girls’ thoughts and opinions on having social media influencers deliver body image and mental health support: A mixed-methods study by Sharon Haywood, Phillippa C Diedrichs and Nicole Paraskeva in DIGITAL HEALTH

sj-docx-6-dhj-10.1177_20552076251361340 - Supplemental material for Understanding adolescent girls’ thoughts and opinions on having social media influencers deliver body image and mental health support: A mixed-methods study Supplemental material, sj-docx-6-dhj-10.1177_20552076251361340 for Understanding adolescent girls’ thoughts and opinions on having social media influencers deliver body image and mental health support: A mixed-methods study by Sharon Haywood, Phillippa C Diedrichs and Nicole Paraskeva in DIGITAL HEALTH

## Notes

1. As the purpose of five close-ended questions and four open-ended questions was to provide the investigators logistical information to help inform future intervention development (e.g. preferred types of vlogs, names of influencers girls follow, and the ideal length of a vlog), these results are not reported on in this article.
