# Supplementary material for: Understanding adolescent girls’ thoughts and opinions on having social media influencers deliver body image and mental health support: A mixed-methods study
Source: Digit Health. 2025 Aug 3;11:20552076251361340. doi: 10.1177/20552076251361340 (PMC12319284; doi:10.1177/20552076251361340)
Supplement: sj-docx-1-dhj-10.1177_20552076251361340 - Supplemental material for Understanding adolescent girls’ thoughts and opinions on having social media influencers deliver body image and mental health support: A mixed-methods study [file sj-docx-1-dhj-10.1177_20552076251361340.docx]

**Supplementary Material 1: Complete survey**

**Demographics^[[1]](#footnote-1)^**

1. **What is your / your daughter’s current age?**
   - 13
   - 14
   - 15
   - 16
   - 17
   - 18
2. **How would you describe your / your daughter’s ethnic origin?**

- Arab
- Bangladeshi
- Black African
- Black British or any other black background
- Black Caribbean
- Chinese
- Indian
- Mixed origin/multiple ethnic background - White & black Caribbean
- Mixed white and Asian
- Mixed white and black African
- Other Asian background
- Other mixed/multiple ethnic background
- Other white background, e.g. European, American etc.
- Pakistani
- White English / Welsh / Scottish / Northern Irish / British
- White Irish
- Any other ethnic group_____
- Prefer not to say
- Don’t know

**3. Please indicate which region of the UK you are resident of?**

- Greater London
- South East
- South West
- West Midlands
- North West
- North East
- Yorkshire & Humberside
- East Midlands
- East Anglia
- Wales
- Scotland
- Northern Ireland

**4. Please indicate to which occupational group the Chief Income Earner in your household belongs, or which group fits best?**

This could be you: the Chief Income Earner is the person in your household with the largest income.

If you are retired and not reliant on a state pension, please select your occupation before retiring.

Is the Chief Income Earner...

- Manual workers, all apprentices to be skilled trades, Caretaker, Park keeper, non-HGV driver, shop assistant
- Skilled manual worker (e.g., Skilled Bricklayer, Carpenter, Plumber, Bus/ Ambulance Driver, HGV driver, AA patrolman, pub/bar worker, etc)
- Supervisory or clerical/ junior managerial/ professional/ administrative (e.g., Office worker, Student Doctor, Foreman with 25+ employees, salesperson, etc)
- Intermediate managerial/ professional/ administrative (e.g., Newly qualified (under 3 years) doctor, Solicitor, Board director small organisation, middle manager in large organisation, principal officer in civil service/local government)
- Higher managerial/ professional/ administrative (e.g., Established doctor, Solicitor, Board Director in a large organisation (200+) employee, top level civil servant/public service employee)
- Student
- Casual worker not in permanent employment
- Housewife/ Homemaker
- Retired and living on state pension (If you are retired, and not living on state pension, please select your occupation before retiring)
- Unemployed or not working due to long-term sickness
- Not working due to disability
- Full-time carer of another household member

**Participant Informed Assent**

Thank you for your interest in taking part in this research project!

Before you decide whether to take part, we want to make sure that you understand why the study is being done and what it will involve. Please read the following information carefully, and if you have any questions or would like more information, please let us know.

**Who is organising the research?**

The work is being carried out by Dr Nicole Paraskeva and Sharon Haywood who are researchers based at the Centre for Appearance Research at the University of the West of England.

**What is the aim of the research?**

We are interested in creating a series of vlogs (video blogs) on the topics of wellbeing and body image for young people like yourself, so we would like your opinions. To do this we would like to ask you about your experience of social media influencers and vlogs, and to find out what you think makes vlogs interesting (or not!).

**What do I have to do?**

If you choose to take part in the study, we will ask you to complete an online survey that should take no more than 20 minutes to complete. We will ask you questions about your social media use and social media influencers (for example, who you follow on social media and why). We are also interested in what you like and dislike when it comes to content that social media influencers share. As well, we will explore what you think about influencers delivering content to help young people improve their body image. Your parent/guardian has already provided their permission for you to participate in this study. If you would like to participate, we will ask you to confirm that you are happy to participate before starting the survey. As a thank you for your time, you will receive points through the research agency that you can use for a reward,

such as a voucher.

**Do I have to take part?**

No – your participation is voluntary, so you do not have to take part in this study if you do not want to. You have the right to stop taking part in the study at any point without having to say why. You can tell the researcher or your parent/guardian if you would like to stop participating. If you choose to complete the survey and then decide that you no longer want us to use your survey answers in

our research, let us know and we will remove it from the study. We can remove your data up to two weeks after the survey. After this time, your responses will be included in our study.

**What are the benefits of taking part?**

By understanding your views on this topic, the researchers hope to develop vlogs that will be delivered by social media influencers that will be most effective in improving young people’s wellbeing and body image.

**What are the possible risks of taking part?**

There are very minimal risks involved. We will not ask you any information about your own wellbeing or body image. However, just in case you find answering general questions about influencers upsetting, we will provide you with details of sources of support through organisations websites and helplines if you need them after you take part.

**What will happen to my information?**

We will keep your answers private. Responses you give us during the research study will be confidential and you will not be identified by anyone else. Any personal information that could identify you will be removed or changed before data is shared with other researchers outside the UWE Bristol team or reports or other written materials about the study are published.

**Contact information**

If you have questions or would like more information, please contact [Nicole.Paraskeva@uwe.ac.uk](mailto:Nicole.Paraskeva@uwe.ac.uk) or [Sharon.Haywood@uwe.ac.uk](mailto:Sharon.Haywood@uwe.ac.uk).

**Are you happy to participate and complete the survey?**

- Yes, I am happy to participate in this research study.
- No, I do not want to participate.

**Introduction**

⭐️ Thanks very much for deciding to take part in this research project! ⭐️

By answering the questions in this survey, it will help us better understand young peoples’ opinions and preferences about social media influencers, and what you think about influencers delivering content to help young people improve their body image.

This survey contains three sections and should take you about 20 minutes to complete.

**Instructions**

For some of the questions, you will need to choose the response that best matches how you feel about a statement or a word. You can only choose one response.

Let’s try an example together.

When you’re not in school or work, how important is it to spend time with your friends?

- Not important at all
- Slightly important
- Somewhat important
- Important
- Extremely important
- If it’s very important that you spend your spare time with friends, you would have chosen “extremely important”.
- If it’s important to spend your spare time with friends most of the time, you would have chosen “important”.
- If it’s important to spend your spare time with friends sometimes, you would have chosen “somewhat important”.
- If it’s important to spend your spare time with friends once in a while, you would have chosen “slightly important”.
- If it’s not important at all to spend your spare times with friends, you would have chosen “not important at all”.

Do you understand how to answer this type of question?

- Yes, I’m ready to start.
- No, I’m not sure.

**If “no” was selected:**

Please contact [redacted], Director at QRS Market Research at [redacted], for help understanding how to answer the questions. After speaking with [redacted] and you’re clear on how to complete the questions, please click “Next” to begin.

**Social media use**

Okay, let's begin!  🚀

How many hours each week do you spend on YouTube?

- Less than one hour
- 1-2 hours
- 2-4 hours
- 4-6 hours
- 6-8 hours
- 8-10 hours
- More than 10 hours

How many hours each week do you spend on TikTok?

- Less than one hour
- 1-2 hours
- 2-4 hours
- 4-6 hours
- 6-8 hours
- 8-10 hours
- More than 10 hours

**SECTION A: INFLUENCERS & SOCIAL MEDIA**

This section contains ten (10) questions about your opinions about influencers on social media. Please note that “social media influencers” are sometimes called “content creators”.

1. **When deciding to watch or follow social media influencers, how important are the following influencer qualities?**

|  | Not important at all | Slightly important | Somewhat important | Important | Extremely important |
| --- | --- | --- | --- | --- | --- |
| - - - 1. Authenticity (being genuine and real) | 1 | 2 | 3 | 4 | 5 |
| - - - 1. Likeability | 1 | 2 | 3 | 4 | 5 |
| c. Credibility (being believable) | 1 | 2 | 3 | 4 | 5 |
| d. Relatability (feeling like you can identify) | 1 | 2 | 3 | 4 | 5 |
| e. How similar influencers are to me (appearance, background, interests) | 1 | 2 | 3 | 4 | 5 |

1. **How much do you agree with the following statements?**

|  | Totally disagree | Mostly disagree | Neither agree nor disagree | Mostly agree | Totally agree |
| --- | --- | --- | --- | --- | --- |
| 1. I listen to/take the advice of influencers I follow if they suggest an activity (e.g., trying a new type of sport). | 1 | 2 | 3 | 4 | 5 |
| 1. I listen to/take the advice of influencers I follow who provide information (e.g., about an exercise class). | 1 | 2 | 3 | 4 | 5 |

1. **Name your favourite three YouTubers and explain why you watch them:**
   - _______________________________________________________________
   - _______________________________________________________________
   - _______________________________________________________________
2. **Name your favourite three influencers on TikTok and explain why you watch them:**
   - _______________________________________________________________
   - _______________________________________________________________
   - _______________________________________________________________
3. **Which of the following YouTubers have you watched and enjoyed their content? Please choose all that apply.**
   - Anna Archer
   - Demi Donnelly
   - Leena Norms
   - Emily Philpott
   - Charlotte Emily Price
   - Tiana Wilson
   - Lucy Wood
   - None of the above
4. **Which of the influencers on TikTok have you watched and enjoyed their content? Please choose all that apply.**
   - Miah Carter
   - Summer Fox
   - Millie McLay
   - Florence Simpsonn
   - Tennessee Thresher
   - None of the above
5. **What types of vlogs/videos made by influencers do you enjoying watching? Please choose all that apply:**
   - “A day in my life”
   - “Get ready with me”
   - “How to” videos
   - Current affairs
   - Morning/night routine
   - Trending topics
   - Other (please describe): ________________
6. **When listening to/taking the advice of an influencer, how important is it that the following characteristics of the influencer are similar to you?**

|  | Not important at all | Slightly important | Somewhat important | Important | Extremely important |
| --- | --- | --- | --- | --- | --- |
| 1. Ethnicity (i.e., they’re the same ethnicity as you) | 1 | 2 | 3 | 4 | 5 |
| 1. Gender (i.e., they’re the same gender as you) | 1 | 2 | 3 | 4 | 5 |
| 1. Age (i.e., they’re a similar age to you) | 1 | 2 | 3 | 4 | 5 |
| 1. Body size i.e., they’re a similar body size to you | 1 | 2 | 3 | 4 | 5 |

1. **Who would you be more likely to listen to and take advice from? (select one)**
   - An influencer younger than me
   - An influencer the same age as me
   - An influencer a few years older than me
   - An influencer much older than me
   - It doesn’t matter
2. **What device do you use MOST OFTEN to access social media? Please choose ONE response:**
   - Desktop computer
   - Laptop
   - Tablet
   - Mobile phone

Brilliant! You have completed half the survey!


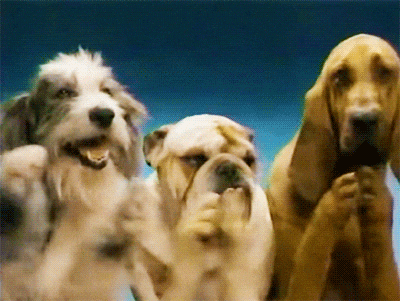


**SECTION B: BODY IMAGE**

This second section contains some general questions about body image, which refers to **how you think and feel about your body, including how it looks and what it can do**. There are six (6) questions in this section.

1. **If you wanted help with your body image, how likely are you to do the following?**

|  | Extremely unlikely | Unlikely | Neutral | Likely | Extremely likely |
| --- | --- | --- | --- | --- | --- |
| a. Look for help regarding body image issues online (e.g., social media, websites, forums)? | 1 | 2 | 3 | 4 | 5 |
| b. Look for help regarding body image issues offline (face-to-face, doctor, school counsellor)? | 1 | 2 | 3 | 4 | 5 |

1. Please explain your answers to the above two questions: ______________________________________________________________________________________________________________________________________________________________________________________________________________________________
2. **Have you looked for information on social media or elsewhere related to body image? Please choose all that apply:**
   - Instagram
   - TikTok
   - YouTube
   - Facebook
   - Twitter / X
   - Twitch
   - Snapchat
   - Other social media platform
     1. What is the name of the platform(s)? __________________________
   1. Other:
      1. Please describe where else you have looked for information: ____________________________________________
3. **When trusting and taking the advice of an influencer about body image, how important are the following factors?**

|  | Not important at all | Slightly important | Somewhat important | Important | Extremely important |
| --- | --- | --- | --- | --- | --- |
| 1. The influencer has shared their personal experiences with body image. | 1 | 2 | 3 | 4 | 5 |
| 1. The number of followers they have. | 1 | 2 | 3 | 4 | 5 |
| 1. The influencer working with an expert (e.g., psychologist) in body image. | 1 | 2 | 3 | 4 | 5 |
| 1. The influencer working with a company or business to share tips and tools on body image and mental health. | 1 | 2 | 3 | 4 | 5 |

1. **What type of influencers do you think would be best to deliver vlogs featuring tips and techniques to improve body image and wellbeing? Please choose all that apply:**
   1. Beauty and Fashion
   2. Sports and Fitness
   3. Wellbeing
   4. Mental health
   5. Travel
   6. Lifestyle
   7. Tech and Gaming
   8. Cooking
   9. Other (please describe):___________
2. **Which social media platform(s) do you think would be BEST for mid- to long-form content (approximately 10 minutes or longer) containing tips and techniques to improve body image? Please choose ONE response:**
   - Instagram
   - TikTok
   - YouTube
   - Facebook
   - Twitter / X
   - Snapchat
   - Other social media platform
   - What is the name of the platform(s)? __________________________
3. **We would like to explore using influencers to deliver vlogs/videos to young people like yourself about how to improve body image.**

a) Do you think this is a good idea to help improve young people’s body image?

| Totally disagree | Mostly disagree | Neither agree nor disagree | Mostly agree | Totally agree |
| --- | --- | --- | --- | --- |
| 1 | 2 | 3 | 4 | 5 |

b) Please explain your answer: ______________________________________________________________________________________________________________________________________________________________________________________________________________________________

1. **If we create a series of 3-5 vlogs/videos on body image, how long do you think EACH vlog/video should be? Please choose ONE response:**
   1. Less than a minute
   2. 1-5 minutes
   3. 6-10 minutes
   4. 11-15 minutes
   5. 16-20 minutes
   6. More than 20 minutes

**SECTION C: MENTAL HEALTH AND WELLBEING**

This final section has four (4) questions about **mental health and overall wellbeing**. For example, this might relate to depression or anxiety.

**1. What do you think are the BEST ways to reach teenagers with support, advice, and programmes to help them with their wellbeing and mental health? Please choose all that apply:**

- School workshops
- Posters or pamphlets in a GP surgery
- TV campaigns
- Adverts on social media
- Social media influencers sharing good quality advice and tips
- NHS website
- Books
- Podcasts
- Counsellors available at schools
- Telephone support lines
- Specific charities and organisations
- Other (please specify): ____________________

1. **In your opinion, what are the advantages or positives of influencers delivering content to improve wellbeing and mental health, if any?**

__________________________________________________________________________________________________________________________________________________________________________________________________________________________________________________________________________________________________________________________________________________________________________________________________________________________

1. **In your opinion, what are the disadvantages or negatives of influencers delivering content to improve wellbeing and mental health, if any?**

__________________________________________________________________________________________________________________________________________________________________________________________________________________________________________________________________________________________________________________________________________________________________________________________________________________________

1. **What are your overall thoughts and opinions on influencers delivering content related to improving wellbeing and mental health?**

__________________________________________________________________________________________________________________________________________________________________________________________________________________________________________________________________________________________________________________________________________________________________________________________________________________________

Thank you for your participation and helping us with this important research!

**
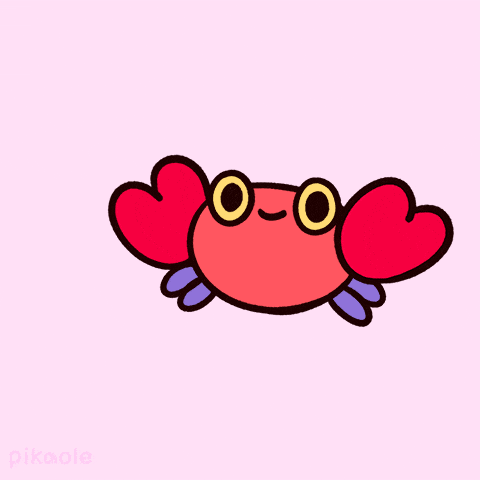
**

Please click “Next” to close the survey ➡️

1. Demographics were completed by 18-year-old participants and the parents/guardians of participants under the age of 18. [↑](#footnote-ref-1)
