# Supplementary material for: Understanding adolescent girls’ thoughts and opinions on having social media influencers deliver body image and mental health support: A mixed-methods study
Source: Digit Health. 2025 Aug 3;11:20552076251361340. doi: 10.1177/20552076251361340 (PMC12319284; doi:10.1177/20552076251361340)
Supplement: sj-docx-3-dhj-10.1177_20552076251361340 - Supplemental material for Understanding adolescent girls’ thoughts and opinions on having social media influencers deliver body image and mental health support: A mixed-methods study [file sj-docx-3-dhj-10.1177_20552076251361340.docx]

**Supplementary Material 3.** Content analysis of subcategories of participants’ open-text responses explaining participants’ likelihood of seeking body image support online and offline (questions B1a and B2a). Neutral responses are not included in the table^1^.

| **Likelihood of seeking body image support online**  **(*N* = 382 responses)** | | | | **Likelihood of seeking body image support offline**  **(*N* = 379 responses)** | | | |
| --- | --- | --- | --- | --- | --- | --- | --- |
| **Likert scale response** | **Subcategory** | **Frequency**  ***n* (%)** | **Example quote** | **Likert scale response** | **Subcategory** | **Frequency**  ***n* (%)** | **Example quote** |
| **Likely^2^**  **(*n* = 183; 48%)** | Can obtain general advice and support | 58 (31.7) | “google is always good for finding things out” | **Likely^2^**  **(*n* = 147; 38.8%)** | Trust and feel confident dealing face-to-face with professionals | 65 (44.2) | “I feel that I can talk to professional people with confidence” |
|  | Can learn from other people with the same issue(s) | 28 (15.3) | “Can find many relatable people that have gone through a similar experience and give advice” |  | It's easier / more appropriate | 25 (17.0) | “Easier to talk to in the real world” |
|  | Provides easy access | 20 (10.9) | “It’s easy to find stuff online.” |  | Prefer to receive support from family and/or friends | 24 (16.3) | “I am more likely to ask friends and family” |
|  | Would use to learn about exercises and diets | 18 (9.8) | “I can get information about fitness, types of exercise, what to eat” |  | Can get personalised help | 4 (2.7) | “I get more personalised in depth advice” |
|  | Provides anonymity | 12 (6.6) | “I would like to get ideas without having to talk to someone” |  | Don’t know / not sure | 4 (2.7) | “not sure” |
|  | Influencers are inspiring | 4 (2.2) | “Because they are inspo” |  | Miscellaneous | 16 (10.9) | “I go to consoling [sic] every week” |
|  | Miscellaneous | 28 (15.3) | “good place to look” |  | No text response | 9 (6.1) | n/a |
|  | No text response | 15 (8.2) | n/a |  |  |  |  |
| **Unlikely^3^**  **(n = 111; 29%)** | Don’t have body image issues/not interested | 30 (27.0) | “Because I am happy with my body” | **Unlikely^3^ (n = 144; 38%)** | Embarrassed / wants anonymity | 69 (47.9) | “I might feel embarrassed talking to someone I know” |
|  | Unrealistic beauty standards online | 29 (26.1) | “because social media is fake and only a few people share what their body really looks like” |  | Don’t need help | 24 (16.6) | “I like the way I am” |
|  | Prefer to speak with family or friends | 21 (18.9) | “I would speak to my mum mainly instead of social media” |  | General negative comments | 20 (13.9) | “I won’t be taken seriously” |
|  | Prefer doctor or professional | 4 (3.6) | “I would ask my dr” |  | Prefer friends and/or family | 5 (3.5) | “My dad and teachers are always happy to help and one of my friends suffers from an eating disorder so my dad checks up on me frequently, which isn't a bad thing.... Just a little annoying but he loves me so I am OK with it.” |
|  | Could receive incorrect information | 6 (5.4) | “Because people online may give bad advice” |  | Online is preferred/easier | 5 (3.5) | “Would be easier to do it online than face to face with someone” |
|  | I don’t know / not sure | 3 (2.7) | “I don’t know never thought of that” |  | Inconvenient | 4 (2.8) | “Not confident and can never get a doctors appointment” |
|  | Miscellaneous | 16 (14.4) | “I am who I am” |  | Don’t know / not sure | 4 (2.8) | “unsure” |
|  | No text response | 2 (1.8) | n/a |  | Miscellaneous | 7 (4.9) | “they make me look cool” |
|  |  |  |  |  | No text response | 6 (4.2) | n/a |

^1^ Neutral responses for participants’ likelihood of seeking online body image support accounted for 23% (*n* = 88). Neutral responses for participants’ likelihood of seeking offline body image support accounted for 23.2% (*n* = 88).

^2^Includes “likely” and “extremely likely” Likert-scale responses.

^3^Includes “unlikely” and “extremely unlikely” Likert-scale responses.
