# Supplementary material for: Understanding adolescent girls’ thoughts and opinions on having social media influencers deliver body image and mental health support: A mixed-methods study
Source: Digit Health. 2025 Aug 3;11:20552076251361340. doi: 10.1177/20552076251361340 (PMC12319284; doi:10.1177/20552076251361340)
Supplement: sj-docx-4-dhj-10.1177_20552076251361340 - Supplemental material for Understanding adolescent girls’ thoughts and opinions on having social media influencers deliver body image and mental health support: A mixed-methods study [file sj-docx-4-dhj-10.1177_20552076251361340.docx]

**Supplementary Material 4.** Content analysis subcategories of open-text responses (*N* = 376) explaining participants’ agreement or disagreement with using influencers to deliver vlogs/videos to young people about how to improve body image (question B5a). Neutral responses are not included in the table^1^.

| **Likert scale response** | **Subcategory** | ***n* (%)** | **Example quote** |
| --- | --- | --- | --- |
| **Agree^2^**  **(*n* = 230; 61.2%)** | Helpful for health and wellbeing | 52  (22.6) | “To improve the overall mental health of young people” |
|  | Body image is an important issue | 29 (12.6) | “It is more common in the younger generation for body insecurity and this needs to be explicitly addressed” |
|  | General comments in agreement | 26 (11.3) | “Think it would be good for young people to see” |
|  | The internet is a good vehicle to reach young people | 23  (10.0) | “It can help those who can’t get to any doctors and it’s easy as they can also show tutorials” |
|  | Influencers are relatable | 22  (9.5) | “people my age will be able to relate to them” |
|  | Influencers are trustworthy | 13 (5.7) | “I selected totally agree because influencers are very relatable with me and I also trust their opinions and advice” |
|  | Influencers can relate to body image issues | 8 (3.5) | “They would have been in our position before and know what we are going through.” |
|  | Can increase confidence | 8 (3.5) | “It will help them feel more confident with their body down the line” |
|  | Can increase media literacy skills | 3 (1.3) | “Because so many people go on about weight and body type and people edit pictures which makes you feel bad about yourself” |
|  | Vlogs would be interesting and inspiring | 3 (1.3) | “Because young people like myself would watch and be interested to find out more from these vlogs and videos” |
|  | Not sure | 3 (1.3) | “I don’t know” |
|  | Miscellaneous | 27 (11.7) | “A good body is a healthy body” |
|  | No text response | 13 (5.7) | n/a |
| **Disagree^3^**  **(*n* = 51; 13.6%)** | Influencers are untrustworthy | 10 (19.6) | “I wouldn’t trust their opinions” |
|  | Could cause harm | 10 (19.6) | “Unless they are a professional their advise [sic] may be wrong and affect my health” |
|  | Influencers can promote appearance ideals | 11 (21.6) | “It give [sic] young people the wrong idea of how you should look” |
|  | Don’t need help/not interested | 7 (13.7) | “As before I do not require information as I feel confident enough to look after myself” |
|  | One-size-fits-all approach won’t work as we are all different | 3 (5.9) | “Vlogs and videos aren't going to be specialised to an individual person seeking help and advice given may not be relevant” |
|  | Miscellaneous | 10 (19.6) | “It’s their own choice” |

^1^Neutral responses accounted for 25.2% *(n* = 95) of the total responses.

^2^Likert-scale responses include “totally agree” and “mostly agree”.

^3^Likert-scale responses include “totally disagree” and “mostly disagree”.
