# Supplementary material for: Understanding adolescent girls’ thoughts and opinions on having social media influencers deliver body image and mental health support: A mixed-methods study
Source: Digit Health. 2025 Aug 3;11:20552076251361340. doi: 10.1177/20552076251361340 (PMC12319284; doi:10.1177/20552076251361340)
Supplement: sj-docx-5-dhj-10.1177_20552076251361340 - Supplemental material for Understanding adolescent girls’ thoughts and opinions on having social media influencers deliver body image and mental health support: A mixed-methods study [file sj-docx-5-dhj-10.1177_20552076251361340.docx]

**Supplementary Material 5.** Content analysis subcategories of participants’ open-text responses regarding their views on the advantages and disadvantages of having influencer deliver content to help improve mental health and overall wellbeing (Questions C2 and C3).

| **Advantages^1^**  **(*n* = 263; 77.4%)** | | | **Disadvantages^2^**  **(*n* = 220; 71.9%)** | | |
| --- | --- | --- | --- | --- | --- |
| **Subcategory** | ***n* (%)** | **Example** | **Subcategory** | ***n* (%)** | **Example** |
| Provide help by providing support and information | 79 (30.0) | “That if someone is struggling with their mental health that it’s good to Know that there not alone and that there is help out there for them” | Could deliver incorrect information | 97 (44.1) | “They can influence you the wrong way” |
| Huge reach | 38 (14.4) | “The content will reach wide range of young people easily” | Don’t trust motivations of influencers | 46 (20.9) | “May come across as not genuine especially if they’re being paid to do it and don’t really feel passionate about it” |
| Young people will listen to influencers | 30 (11.4) | “because young people will listen” | Could cause harm | 29 (13.2) | “They could be recommending something that isn't safe. They are not trained professionals so could be doing more harm than good” |
| Young people relate to influencers | 26 (9.9) | “kids can relate to them and will follow what they say” | Influencers promote unrealistic beauty ideals | 11 (5.0) | “Wrong images conveyed of what a body type should be” |
| Influencers can relate to mental health issues and share their personal experiences | 24 (9.1) | “They are reliable cause they mostly sharing [sic] their real experiences” | Mental health issues require personalised attention | 10 (4.5) | “With mental health it is important to receive professional advice face to face” |
| Easy access | 22 (8.4) | “It’s easily accessible and free” | Content will only reach their followers | 7 (3.2) | “Everyone has different preferred influencers, would need a few different styles to catch people” |
| Raise awareness | 12 (4.6) | “To raise awareness and encourage discussion” | Negative commentaries by others online | 7 (3.2) | “The internet can be cruel and make fun of there [sic] influencers affecting there [sic] mental health” |
| Influencers are inspirational | 12 (4.6) | “Since the influences act as role models for young people, them speaking about their wellbeing and mental health might make them feel more relatable, and the influences can act as a source of encouragement and inspiration for their followers to get better.” | Influencers aren’t relatable | 5 (2.3) | “Influencers may not be in touch with the average person and are often not relatable enough” |
| Provide anonymity | 9 (3.4) | “being online so you stay unknown” | Influencer could have bad reputation or be ‘cancelled’ | 4 (1.8) | “An influencer can be ‘cancelled’ for anything so will stop post [sic]” |
| Young people trust influencers | 6 (2.3) | “It’s more likely to be seen and trusted, because you just ignore what they say at school or parents for this” | Influencers can trigger comparisons | 4 (1.8) | “That you might start to get an inferiority complex” |
| Reduce stigma associated with mental health issues | 5 (1.9) | “takes away any stigma or shame surrounding mental health” |  |  |  |

^1^Total responses (*N* = 340) for the question about advantages included negative comments (9.4%; *n* = 32) and neutral responses (13.2%; *n* = 45).

^2^Total responses (*N* = 306) for the question about disadvantages included responses that were neutral or not relevant to the question (i.e., miscellaneous) (28.1%; *n* = 86).
