# Supplementary material for: Understanding adolescent girls’ thoughts and opinions on having social media influencers deliver body image and mental health support: A mixed-methods study
Source: Digit Health. 2025 Aug 3;11:20552076251361340. doi: 10.1177/20552076251361340 (PMC12319284; doi:10.1177/20552076251361340)
Supplement: sj-docx-6-dhj-10.1177_20552076251361340 - Supplemental material for Understanding adolescent girls’ thoughts and opinions on having social media influencers deliver body image and mental health support: A mixed-methods study [file sj-docx-6-dhj-10.1177_20552076251361340.docx]

**Supplementary Material 6.** Content analysis subcategories of participants’ open-text responses (*N* = 361) to question C4 regarding their overall thoughts and opinions on influencers delivering content related to improving wellbeing and mental health.

| **Positive responses**  **(*n* = 238; 65.9%)** | | | **Negative responses**  **(*n* = 48; 13.3%)** | | | **Neutral responses**  **(*n* = 75; 20.8%)** | | |
| --- | --- | --- | --- | --- | --- | --- | --- | --- |
| **Subcategory** | ***n* (%)** | **Example quote** | **Subcategory** | ***n* (%)** | **Example quote** | **Subcategory** | ***n* (%)** | **Example quote** |
| General positive comments | 173 (72.6) | “A positive way of getting information to young people” | General negative comments | 22 (45.8) | “I don't think they should do this. They should stick to entertaining people rather than trying to change them” | Unsure | 31 (41.3) | “I think it’s a bad and a good idea at the same time” |
| Could be useful in conjunction with experts | 26 (10.9) | “It can be positive if done professionally and they are trained to give the right advice” | Could cause harm | 8 (16.7) | “Confusing and dangerous is [sic] gives teenagers like me bad and possibly unhealthy advice” | Not interested / no opinion | 25 (33.3) | “not a big fan I just want to watch funny videos” |
| Could be a good approach if done right (non-specified) | 19 (8.0) | “It’s good if done in a positive way” | Influencers lack appropriate training | 7 (14.6) | “They are not trained so could go wrong” | Depends on the influencer’s motivation and/or content | 16 (21.3) | “If it was a good person then yes but if it’s a clout chaser it would feel fake” |
| Could be good if the influencer was genuine | 8 (3.4) | “I think its [sic] great, as long as he is honest” | Don’t like and/or trust influencers | 6 (12.5) | “Okay but I would not believe them” | Miscellaneous | 3 (4.0) | “If it helps people great people don’t need to click and watch” |
| Could be good if the influencer has had experience with the mental health issue | 8 (3.4) | “Could be a good thing but only if they speak from experience” | Influencers may be motivated by money | 5 (10.4) | “Most of them are just business to make money” |  |  |  |
| Could be useful in conjunction with offline support | 4 (1.7) | “My overall thoughts is that influencers can be a useful complement to professional help” |  |  |  |  |  |  |
